# *CLN5* in heterozygosis may protect against the development of tumors in a VHL patient

**DOI:** 10.1186/s13023-020-01410-y

**Published:** 2020-06-02

**Authors:** Isabel de Rojas-P, Virginia Albiñana, Lucía Recio-Poveda, Amanda Rodriguez-Rufián, Ángel M. Cuesta, Luisa-María Botella

**Affiliations:** 1 Centro de Investigaciones Biológicas Margarita Salas, Consejo Superior de Investigaciones Científicas (CSIC), Madrid, Spain; 2 Centro de Investigación Biomédica en Red de Enfermedades Raras (CIBERER), group U707, 28040 Madrid, Spain

**Keywords:** Von Hippel-Lindau (VHL), Neuronal Ceroid Lipofuscinosis type 5 (CLN5), Rare disease, Hemangioblastoma (HB), Clear cell renal cell carcinoma (ccRCC), Endothelial cells (ECs)

## Abstract

Von Hippel-Lindau syndrome (VHL) is a rare disease of dominant inheritance that increases susceptibility to tumor development, with a complete penetrance at the age of 60. In this report, we present the unprecedented case of a VHL carrier who remains healthy at 72. Under the course of this study, it was discovered that this patient carries a mutation for a second rare disease, Neuronal Ceroid Lipofuscinosis (NCL or CNL). We hypothesize that the *CLN* mutation she carries offers a protective effect, preventing tumor development in the cells potentially suffering a *VHL* second hit mutation. To test this hypothesis, we ran a series of molecular experiments and confirmed that cell viability of primary endothelial cells decreases upon *CLN5* silencing. Our results further elucidate the cell biology implications of two rare diseases interacting.

## Introduction

A rare disease affects, by definition, no more than 1 in 2000 individuals in the European Union [1], and less than 200,000 total in the United States [2]. Although the likelihood of having one rare disease is small, more than 7000 different types have been described, accounting for 30 million patients in Europe alone [3–5]. Despite the increasing efforts to investigate rare diseases [6], little is known about families, or patients carriers or suffering more than one rare condition. The co-occurrence/segregation of two independent diseases in consanguineous and non-consanguineous families has been reported in the past [7], but the molecular interaction between the conditions has seldom been investigated. In this case report we present a unique and, to the best of our knowledge, unprecedented combination of two rare diseases segregating in a family, leading to an unexpected clinical picture: a 72 year old woman carrying mutations in heterozygosis for both Neural Ceroid Lipofuscinosis (NCL or CNL) and Von Hippel Lindau disease (VHL), with no development of any pathology.

NCL are a group of neurodegenerative disorders of recessive inheritance, with an incidence as high as 1 in 12,500 births [8]. Currently, fourteen genetically differing forms have been described, corresponding to mutations in different genes (*CLN1* to *CLN14*), and they are all characterized by loss of nerve cells and accumulation of lipopigments within cells. This lysosomal lipid accumulation is due to an inability to degrade lipids, and leads to a neurological atrophy typical of the disease [9, 10]. The patient of this case report is NCL symptom-free, as she is a carrier in heterozygosis for a *CLN5* mutation.

The other rare disease in play is VHL, a dominant autosomal disorder affecting 1 in every 36,000 births, characterized by the susceptibility to a series of tumors, typically hemangioblastomas (HB) of the Central Nervous System (CNS) or retina, clear cell renal cell carcinomas (ccRCC) and pheochromocytomas [11]. These develop after a second hit mutation in *VHL -* a tumor suppressor gene - causes the loss of functional VHL protein [12, 13]. Under normoxic conditions, VHL protein recognizes and binds the previously hydroxylated Hypoxia Inducible Factor (HIF) to trigger its proteasomal degradation [14]. Tissues suffering a stochastic VHL second hit mutation unfold a lack of functional VHL protein, which induces a state of pseudo-hypoxia, promoting tumor growth in these tissues where cells have lost heterozygosis [15].

Despite VHL’s dominant inheritance and almost complete penetrance at the age of 60 [16–18], the patient here presented has not shown any VHL symptoms throughout her lifetime. However, her son inherited her mutation and developed bilateral suprarenal tumors in his thirties. Given the family history of two rare diseases, this led us to think of a possible interaction between NCL and VHL. In order to determine the possibility of said interaction, we completed the genetic screening of the patient and her relatives, and performed cellular and molecular assays on primary and established cell lines.

The combination of our in vitro results and the clinical data gathered from the studied family points towards a protective effect by NCL in this patient regarding tumor development: VHL cells that suffer a second hit mutation in *VHL* cannot divide and progress to develop a tumor, due to the lower viability caused by NCL haplo-insufficiency, interfering in some way with the process of tumorigenesis. These data show a unique counteracting interaction resolving in a symptom-free patient.

## Results and discussion

### Background: family history

**Fig. 1:**
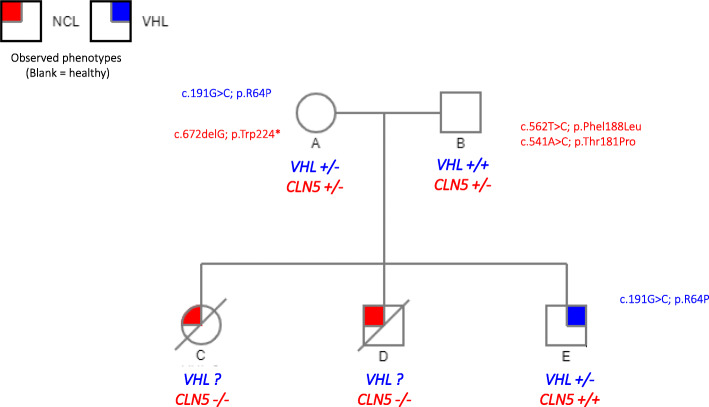
Genetic pedigree of the family of interest showing information on their VHL and CLN5 genotypes and phenotypes (healthy, lipofuscinosis affected or VHL). Circles represent females and squares represent males. The genotype and phenotype of each family member is indicated underneath. Subject A is the subject of interest carrying a *VHL* mutation and not developing any tumors. Black arrow indicates first family member diagnosed with VHL

The family here presented came to our attention through our collaboration with the Spanish VHL patient Alliance. The first member of the family to be diagnosed with VHL was subject E (Fig. 1), who presented with bilateral pheochromocytomas at the age of 34. Upon genetic screening of the immediate relatives, it was discovered that subject A carried the same *VHL* mutation as subject E, and thus had been maternally transmitted to him.

Intriguingly, subject A remains completely healthy at the age of 72, despite her *VHL* mutation. Since her diagnosis, she undergoes annual examinations according to the international follow-up protocol for VHL disease, which includes direct and indirect ophthalmoscopy, MRI of the CNS, abdominal MRI, diagnostic audiologic evaluation and catecholamines tests. No clinical findings of VHL have been found so far, constituting the only known case to the best of our knowledge, of a VHL patient lacking any of the disease symptoms. Taking a closer look at the family’s history, we discovered that patient A had two elder sons who died as teenagers, due to a different rare disease: NCL. Upon learning this, we realized that patient A is carrier of a *CLN* mutation, in particular at the *CLN5* gene.

Altogether, the family’s history suggests that her lack of VHL symptoms can be based on a protective effect that would prevent tumor development. At this point our hypothesis was that this subject’s cells, in case of suffering a *VHL* second hit mutation normally leading to tumor formation [11, 12], would be no longer viable due to their *CLN5* mutation, and thus would not progress into tumor. The next experiments were designed to test this hypothesis.

### *CLN5* silencing reduces cell viability in different primary and established cell lines

#### Hemangioblastoma primary cell culture

**Fig. 2:**
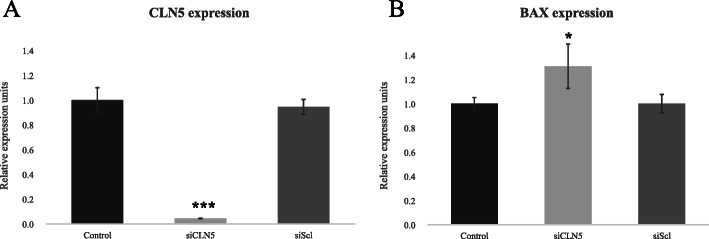
qPCR results in HB primary cells, 72 h after transfection with siCLN5 and siScrambled (siScl)*.* Control group was left untreated, while siScl serves as a negative control. The experiment was conducted in triplicates, representing the mean and showing the standard deviation as error bars. **a***CLN5* expression; **b***Bax* expression. Statistical significance is represented as *p < 0.05; **p < 0.01; and ***p < 0.001

**Fig. 3:**
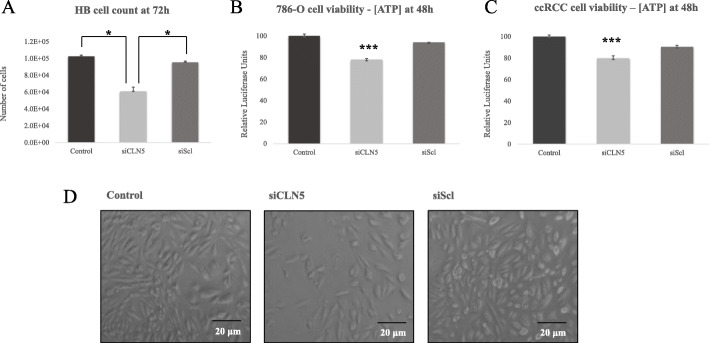
**a** Cell count by Bio-Rad TC20 Automated Cell Counter. Primary HB cells were trypsinized 72 h after transfection with siCLN5 and siScl as negative control. Cell viability was measured in 786-O cells **(b)** and ccRCC primary cells **(c)** 48 h after transduction by a luciferase assay, where ATP levels are a direct correlation of cell viability. **d** ccRCC primary cell cultures under the microscope were photographed 72 h after transfection. Control group was left untreated, while siScl serves as negative control. Every condition was replicated three times – the graphs show the combined mean of these experiments, representing the obtained standard deviation in the error bars. Statistical significance is represented as *p < 0.05; **p < 0.01; and ***p < 0.001

To test our hypothesis, we started by silencing *CLN5* in HB cells from a VHL patient. These cells have already suffered the second hit mutation and thus are *VHL* (−/−), adequate to test how a CLN5 protein deficiency might affect cell viability in them. In vitro cell cultures showed a significantly reduced number of cells in the *CLN5* transfected groups, quantitatively supported by cell counter measurements (Fig. 3a). Next, to assess if apoptosis was promoting cell death in siCLN5 transfected cells, *Bax* gene expression was quantified by quantitative PCR (qPCR). *Bax* was chosen for its known involvement in triggering apoptosis [19]. RT-qPCR analysis showed that *CLN5* silencing was successful (Fig. 2a), and that the levels of pro-apoptotic gene *Bax* appeared significantly elevated in the partially *CLN5* silenced population (Fig. 2b). These results taken together make us think that CLN5 deficient HB cells have a disadvantage and do not expand at the rate control groups do, since their viability is compromised.

#### 786-O cell culture

As primary cultures of HB are sensitive to the process of transfection by liposomes and were not expanding optimally, we chose to continue with a more resistant *VHL* (−/−) sample, the human ccRCC 786-O cell line. This time, as a direct measure of cell viability, we quantified ATP levels at different times after *CLN5* gene silencing. The reduction in cell viability was best seen at 48 h, with a 20% loss compared to control (Fig. 3b). The concordance between the positive and negative controls shows that the transfection reagent used for silencing (Lipofectamine) does not have a significant effect on cell viability, and therefore, the drop in cell viability in partially silenced *CLN5* cells is due only to the decrease of CLN5 transcript levels.

#### ccRCC primary cell culture

Although we observed a decrease in cell viability upon CLN5 depletion, this effect might be diluted by the high replicative rate characteristic of a cell line. This is why we chose a second *VHL* (−/−) primary cell culture, different than HB (which was not expanding optimally): primary ccRCC cells derived from the tumor of a VHL patient. The previous results were successfully replicated – again, a 20% decrease in cell viability is observed in the si*CLN5* group, 48 h after transfection (Fig. 3c). This was visually apparent under the microscope; *CLN5* silenced cells were less confluent than the control groups (Fig. 3d).

### CLN5 protein levels are decreased in endothelial cells derived from VHL patients

Once we established that *CLN5* silencing reduces cell viability in *VHL* (−/−) tumor cells, we wondered whether there is an interaction between CLN5 and VHL, both at transcript and protein levels. We opted for qPCR measurement of *CLN5* gene expression, and Western blotting to determine CLN5 protein levels in endothelial cells (ECs) derived either from VHL patients or from healthy donors. These primary culture cells called BOECs (Blood Outgrowth Endothelial Cells) are heterozygous *VHL* (+/−) in the VHL group, and *VHL* (+/+) in the control group.

**Fig. 4:**
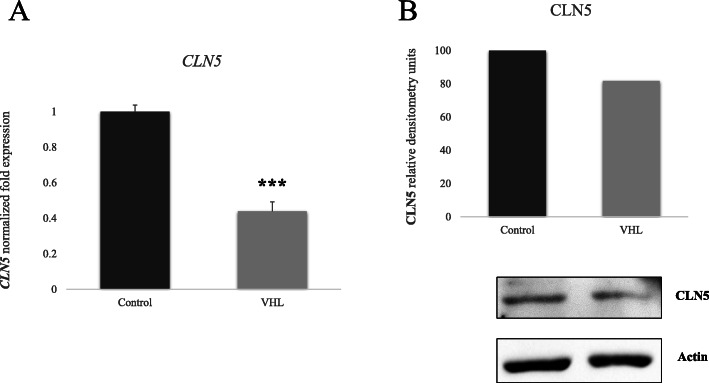
**a***CLN5* expression levels as measured by qPCR. **b** CLN5 and actin protein expression by Western blot and further quantification. Statistical significance is represented as *p < 0.05; **p < 0.01; and ***p < 0.001

Both techniques indicated reduced levels of CLN5 in the VHL group; qPCR showed a 0,6 fold reduction of *CLN5* expression in VHL versus the healthy control (Fig. 4a), and a 20% reduction in CLN5 protein levels in *VHL* (+/−) ECs, compared to control levels (Fig. 4b). These results suggest a putative interaction between CLN5 and VHL: lower levels of VHL protein seem to correlate with a decrease in CLN5 protein expression. We think that there might be a direct interaction of both proteins VHL and CLN5 in the cell, since both are part of the cell degradative pathways - CLN5 is involved in the lysosomal and VHL in the proteasomal degradation. It is possible that a crosstalk among both pathways up to a functioning threshold level may be necessary. Further protein-interaction in vitro experiments would be necessary to confirm this concept.

In summary, subject A, introduced in section 1 and who is *VHL* (+/−) and *CLN5* (+/−), would have a roughly 50% deficiency in CLN5 protein due to her heterozygous *CLN5* mutation. In addition to this, and according to our in vitro results, because of her *VHL* mutation, her cells would be further reduced at CLN5 protein levels. As a result, despite the *CLN5* heterozygous condition, the real CLN5 levels would be below the 50% expected in a heterozygote. Upon a second hit in *VHL*, the levels of CLN5 would fall even lower, below a functional threshold, leading to a pseudolipofuscinosis phenotype in cells suffering this second hit. This condition, highly toxic from a metabolic point of view, would not enable cell proliferation and survival. Thus, the cells suffering a *VHL* second hit mutation will not expand, preventing tumor development. This hypothesis has been supported by an observation made in our lab: BOECs obtained from subject A are not susceptible to *VHL* silencing. After siRNA *VHL* transfection, most of the cells die, and the few remaining have no silenced *VHL*, according to qPCR (data not shown). Overall, our work suggests that a second hit mutation in *VHL* is incompatible with a *CLN5* heterozygosis cellular background. Protective effects of the kind have been previously described in VHL [20], and we propose that this is a similar case, where one mutation suggests the patient to a protection against the disease development.

## Methods

### Cell culture

CNS HB and ccRCC primary cultures from excess of resected surgeries of VHL patients under informed consent were isolated as previously described [21]. HBs, ccRCCs and the human renal cancer cell line 786-O cells (ATCC® CRL-1932™) were all cultured in RPMI supplemented with 20% Fetal Bovine Serum (FBS), 2 mM L-glutamine, and 100 U/mL penicillin/streptomycin (all from GIBCO, Grand Island, NY, USA). All the cellular assays were performed at 37 °C, 5% CO_2_ and ~ 95% humidity.

### Transfection

Cell transfection assays were performed using Lipofectamine® RNAi MAX Reagent (Thermo Scientific, Rockford, IL, USA), following manufacturer instructions. The following siRNAs were used: Scrambled (#SIC001) from Sigma-Aldrich (Saint Louis, MO, USA) for control, and a combination of 1-CLN5 (#8149), 2-CLN5 (#18061) and 3-CLN5 (#146678) from Ambion/Thermo Scientific for *CLN5* silencing. The transfection efficiency was around 75%, as estimated by experiments with fluorescent probes conducted in parallel.

### Cell viability assay

The viability of the ccRCC primary cultures and 786-O cell line was measured by the “Luminescent Cell Viability Assay” (Promega, Madison, WI, USA). This is a homogeneous quantitative method to determine the number of viable cells in culture based on quantitation of the ATP presence, which indicates metabolically active cells. Cells were lysed at 48 h or 72 h after transfection as follows: Cell Titer-Glo reagent (Lysis buffer, Ultra-Glo Recombinant Luciferase, Luciferine, and Mg^2+^) was added to wells to a final proportion of 1:1, and gently mixed for 30 min at room temperature (RT). Next, luminescence was measured in three independent measurements using a Glomax Multidetection System (Promega).

### Real-time RT-PCR (qPCR)

Total RNA was extracted from HB cells using Nucleo Spin RNA kit (Macherey-Nagel, Düren, Germany). One microgram of total RNA was reverse-transcribed in a final volume of 20 μl with the First Strand cDNA Synthesis Kit (Roche, Mannheim, Germany) using random primers. SYBR Green PCR system (BioRad, Hercules, CA, USA) was used to carry out real-time PCR with an iQ5 system (BioRad). Primers used for qPCR are: 18S Fwd: 5′-CTCAACACGGGAAACCTCAC- 3′, 18S Rev.: 5′-CGCTCCACCAACTAAGAACG-3′; BAX Fwd: 5′-CACTCCCGCCACAAAGAT-3′, BAX Rev.: 5′-CAAGACCAGGGTGGTTGG-3′; CLN5 Fwd: 5′-AAGCCCCAGTATGGGAATT-3′, CLN5 Rev.: 5′-TGCCAGTTAATGTACTTCTGAATCC-3′.

### Western blot

For protein extraction, cells were lysed on ice for 30 min in TNE buffer (50 mM Tris, 150 mM NaCl, 1 mM EDTA, and 0.5% Triton X100) supplemented with wide-range protease inhibitors (Roche, Basel, Switzerland) and lactacystin (Sigma-Aldrich), a specific proteasome inhibitor. Lysates were centrifuged at 14,000×g for 5 min. Similar amounts of protein from cleared cell lysates were boiled in SDS sample buffer and analyzed by 4–20% SDS-PAGE under non-reducing conditions (BioRad). Proteins from gels were electro-transferred to nitrocellulose membranes (Amersham, Little Chalfont, UK) followed by immunodetection with anti-CLN5 (Abnova, Taiwan), and anti-actin (Sigma-Aldrich). Following primary antibody incubation overnight at 4 °C, samples were washed and incubated with the corresponding horseradish peroxidase-conjugated secondary antibodies from Dako (Glostrup, Denmark) at RT for 1 h. All antibodies were used at the dilution recommended by the manufacturer. Membranes were developed by chemiluminescence (SuperSignal West Pico Chemiluminescent Substrate, Thermo Scientific).

### Statistical analysis

Results are presented as mean ± SEM. Statistical analyses were performed using the Student’s t-test, comparing siCLN5 results to groups Control and SiScl, independently. Statistical significance was defined when *p* < 0.05 (*p < 0.05; **p < 0.01, ***p < 0.001).

## Data Availability

The datasets used and/or analyzed during the current study are available from the corresponding author on reasonable request.

## Abbreviations

BOECs: Blood Outgrowth Endothelial Cells
ccRCC: Clear cell Renal Cell Carcinomas
CLN or NCL: Neuronal Ceroid Lipofuscinosis
FBS: Fetal Bovine Serum
HB: Hemangioblastoma
HIF: Hypoxia inducible factor
HUVECs: Human Umbilical Vein Endothelial Cells
MRI: Magnetic Resonance Imaging
qPCR: Quantitative Polymerase Chain Reaction
VHL: Von Hippel Lindau
